# A post-GWAS analysis of predicted regulatory variants and tuberculosis susceptibility

**DOI:** 10.1371/journal.pone.0174738

**Published:** 2017-04-06

**Authors:** Caitlin Uren, Brenna M. Henn, Andre Franke, Michael Wittig, Paul D. van Helden, Eileen G. Hoal, Marlo Möller

**Affiliations:** 1 SA MRC Centre for TB Research, DST/NRF Centre of Excellence for Biomedical TB Research, Division of Molecular Biology and Human Genetics, Faculty of Medicine and Health Sciences, Stellenbosch University, Cape Town, South Africa; 2 Department of Ecology and Evolution, Stony Brook University, Stony Brook, New York, United States of America; 3 Institute of Clinical Molecular Biology, Christian-Albrechts-University of Kiel, Rosalind-Franklin-Strasse Kiel, Germany; University of North Carolina at Chapel Hill, UNITED STATES

## Abstract

Utilizing data from published tuberculosis (TB) genome-wide association studies (GWAS), we use a bioinformatics pipeline to detect all polymorphisms in linkage disequilibrium (LD) with variants previously implicated in TB disease susceptibility. The probability that these variants had a predicted regulatory function was estimated using RegulomeDB and Ensembl’s Variant Effect Predictor. Subsequent genotyping of these 133 predicted regulatory polymorphisms was performed in 400 admixed South African TB cases and 366 healthy controls in a population-based case-control association study to fine-map the causal variant. We detected associations between tuberculosis susceptibility and six intronic polymorphisms located in *MARCO*, *IFNGR2*, *ASHAS2*, *ACACA*, *NISCH* and *TLR10*. Our post-GWAS approach demonstrates the feasibility of combining multiple TB GWAS datasets with linkage information to identify regulatory variants associated with this infectious disease.

## Introduction

Genome-wide association studies (GWAS) have advanced the investigation of complex disease genetics and identified thousands of disease-associated variants. This study design compares allele frequencies of common genetic variants across the genome with phenotypic variation in large cohorts of cases and controls. GWAS is based on the premise that causal variants will be in linkage disequilibrium (LD) with the markers present on single nucleotide polymorphism (SNP) arrays. Since 2005, when the first GWAS was published [1], associations have been detected between numerous common genetic variants and several infectious diseases including TB [2–5].

More than 10 GWAS investigating TB susceptibility have been published to date (**Table 1**). These studies investigated the genetic factors associated with TB susceptibility in multiple populations. Thye et al. (2010) performed the first GWAS on TB susceptibility in a case-control cohort from Ghana and the Gambia and identified a region on chromosome 18q11.2 [6]. Within this region, there are numerous immune response genes such as cadherin 13 (*CDH13)*, zinc finger protein 229 (*ZNF229)* and exportin 1 (*XPO1)*. A meta-analysis which included data from Ghana, the Gambia, Russia and Indonesia identified variants at 11p13 that were associated with TB susceptibility [7]. Chimusa et al. (2014) validated several of these loci and identified novel TB associations in a South African case-control cohort [8].

**Table 1:** Previous TB GWAS- results.

Population	Variant/Gene	Number of Cases	Number of Controls	Reference
Ghana	rs4331426 (gene desert)	921	1740	[6]
The Gambia		1316	1382	
Black, White, Asian from USA	rs4893980 *(PDE11A)*	48	57	[9]
	rs10488286 *(KCND2)*			
	rs2026414 *(PCDH15)*			
	rs10487416 (unknown gene)			
Thai and Japanese	Intergenic region between HSPEP1-MAFB	620	1524	[10]
Indonesia	rs1418267 *(TXNDC4)*	108	115	[11]
	rs2273061 *(JAG1)*			
	rs4461087 *(DYNLRB2)*			
	rs1051787 *(EBF1)*			
	rs10497744, rs1020941 *(TMEFF2)*			
	rs188872 *(CCL17)*			
	rs10245298 *(HAUS6)*			
	rs6985962 *(PENK)*			
Ghana	rs2057178 (*WT1*, intergenic )	2127	5636	[7]
The Gambia		1207	1349	
Russia		1025	983	
Indonesia		4441	5874	
South African Coloured	rs2057178, rs11031728 (*WT1*, intergenic)	642	91	[8]
	rs10916338,rs1925714 *(RNF187)*			
	rs6676375 *(PLD5)*			
	rs1075309 *(SOX11)*			
	rs958617 *(CNOT6L)*			
	rs1727757 *(ZFPM2)*			
	rs2505675 *(LOC100508120)*			
	rs1934954 *(CYP2C8)*			
	rs12283022,12294076 *(DYNC2H1)*			
	rs7105967,rs7947821 *(DCUN1D5)*			
	rs6538140 *(E2F7)*			
	rs1900442 *(VWA8)*			
	rs17175227 *(SMOC1)*			
	rs40363 *(NAA60)*			
	rs2837857 *(DSCAM)*			
	rs451390 *(C2CD2)*			
	rs3218255 *(IL2RB)*			
Russia	rs4733781,rs10956514,rs1017281,rs1469288, rs17285138,rs2033059,rs12680942 *(ASAP1)*	5530	5607	[3]
Morocco	rs358793 (Intergenic)	556	650	[12]
	rs17590261 (Intergenic)			
	rs6786408 *(FOXP1)*			
	rs916943 *(AGMO)*			
Uganda and Tanzania	rs4921437 *(IL-12)*	267	314	[13]
Iceland	rs557011, rs9271378 (located between *HLADQA1* and *HLA-DRB1*)	8162	277643	[14]
	rs9272785 *(HLA-DQA1)*			

The majority of TB susceptibility variants previously identified are intronic (S1 Table) and may therefore have some regulatory functions. It has recently become feasible to predict regulatory effects of variants as computational tools, such as RegulomeDB [15] and Ensembl’s Variant Effect Predictor, as well as information regarding the possible impact of regulatory regions have become available [15, 16]. We therefore applied a post-GWAS approach to TB susceptibility to identify possible variants contributing to disease development. A post-GWAS approach entails the use of previous GWAS associations and linkage disequilibrium (LD) data to identify further variants [and possibly the causative variant] that may be associated with the phenotype. This methodology was developed as pinpointing the exact targets of these associations is a challenge [17]. The post-GWAS analysis has previously been used to identify novel functional intronic variants associated with late-onset Alzheimer’s disease [18], cardiovascular disease [19, 20] and human aging [21]. There has been no such analysis on susceptibility to TB.

Here we combine TB GWAS and candidate gene association studies and incorporate knowledge from RegulomeDB [15] and Ensembl’s Variant Effect Predictor to fine-map putative regulatory variants that may predispose an individual to progress to active TB.

## Methods

### Study population

Sample collection was approved by the Health Research Ethics Committee of the Faculty of Health and Medical Sciences, Stellenbosch University (N95/072) and written informed consent was obtained from all study participants. Recruitment was done in two suburbs in the Western Cape, South Africa, where the incidence of TB is high (1340/100 000 population during 1996), although the HIV incidence at the time of sampling was low (~2% of population) [22]. Study participants self-identified as being from the South African Coloured (SAC) population. The admixed SAC population has genetic contributions from five ancestral populations. On average, Bantu-speaking populations contribute ~30%, the KhoeSan ~30%, Europeans 12–18% South Asian ~15%, and East Asian <10% [23–25]. Genotyping of ancestry informative markers (AIMS) was previously performed by Daya et al. (2013). These AIMS were used to infer admixture proportions using ADMIXTURE [25,26].

All study participants were HIV negative and unrelated. TB cases were bacteriologically confirmed (n = 400). The controls had no previous history of TB (n = 366) and were older than 18 years. Tuberculin skin tests (TST) were not performed, as the majority of adults in the communities are TST positive (> 80% of children older than 15 years) [27]. DNA was extracted from blood using the Nucleon BACC3 Kit (Amersham Biosciences, Buckinghamshire, UK).

### Bioinformatics analysis

Data mining was done using the National Health Genome Research Institute’s–European Bioinformatics Institute (NHGRI-EMBI) GWAS catalogue (http://www.ebi.ac.uk/gwas) and PubMed (www.ncbi.nlm.nih.gov/pubmed). Only single nucleotide polymorphisms (SNPs) that were reported to have *p* < 0.05 (after multiple testing correction during association tests) were recorded.

LD was calculated using SNAP [28]. These comparisons were made against Hapmap3 (release 2) data from 4 populations that best represented the ancestral populations of the SAC; Europeans (CEU), Han Chinese (CHB), Luhya (LWK) and Gujarati Indians (GIH). The KhoeSan ancestral component was represented by 2 ≠Khomani genomes. LD between the previously published SNPs and the ancestral genomes was calculated. An *r*^2^ threshold of 0.8 and a window size of 500 kilobases were used as filters. A per population analysis was performed and SNPs were pooled across populations.

**Fig 1:**
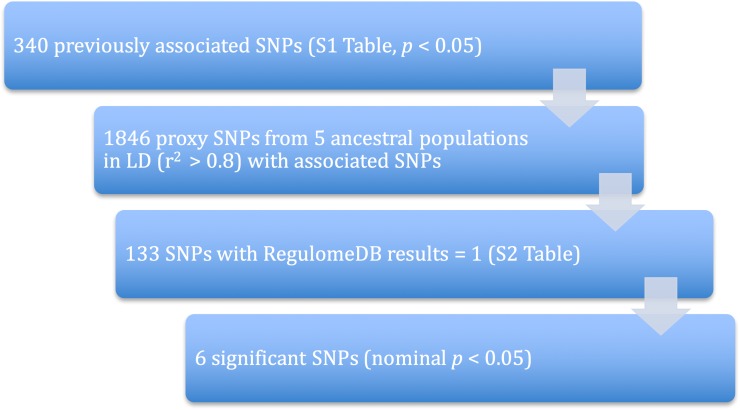
Bioinformatics pipeline for the prioritization of variants.

The potential functional impact of these variants was ascertained by RegulomeDB [15] and Ensembl’s Variant Effect Predictor [16]. Only variants that had a RegulomeDB score of 1 (highest likelihood of a potential functional impact due to the variant) were used in further analysis due to the large number of variants. A summary of the filtering steps used are illustrated in **Fig 1**.

### Genotyping

Genotyping was performed using the Agena MassARRAY^®^ system (Institute of Clinical Molecular Biology, Christian-Albrechts-University, Kiel, Germany). A total number of 133 SNPs were genotyped in 400 cases and 366 controls. Only SNPs that passed quality control (as determined by the confidence in allele call from the Typer Analyzer software package (version 4.0.20, Sequenom proprietary software)) were recorded.

### Statistical analysis

The CaTS power calculator was used to perform power calculations [29]. The power to find a true deviation from the null hypothesis was calculated using a disease allele frequency of 0.06 and an alpha level of 0.05 to determine an odds ratio of 2. A TB disease prevalence of 1% was used in this analysis [30]. With the sample size available, the power to detect a deviation from null was calculated to be 98%.

All statistical analyses were done in R (www.r-project.org) using functions from the base R packages. The Fisher’s exact test was used to calculate Hardy-Weinberg Equilibrium (HWE) *p*-values using functions from the *genetics* R package [31]. Logistic regression models were used to analyse the genotypic and allelic models. All models were adjusted for the confounding factors age, ancestry and sex by including these as covariates. The allelic models (recessive, dominant and additive) were assessed and the model with the highest likelihood to correctly model the data was chosen [32]. Our SNP selection strategy was based on *a priori* evidence that the genes were associated with TB and Bonferroni corrections for multiple testing would be too stringent, risking the rejection of important findings [33–35]. For this reason, the Šidák step-up method was utilized [36]. Nominal *p*-values were corrected for multiple testing using the *multtest* package in R [37] and the cut-off for significance was *p* = 0.05.

## Results

Descriptive statistics were generated for the cases and controls (**Table 2**). Age, sex and KhoeSan ancestry differed significantly between the cases and controls and were therefore all adjusted for in the logistic regression models. Prioritization of SNPs for genotyping is shown in **Fig 1**. Data mining identified 1800 SNPs that were found to be in LD (r^2^ > 0.8) with the 230 SNPs previously associated with TB. After filtering for RegulomeDB scores of 1 (S2 Table), 133 SNPs remained and were genotyped in 400 TB cases and 366 healthy controls. All SNPs were in Hardy-Weinberg Equilibrium (*p* > 0.01). Of the 133 SNPs, 6 variants were found to be statistically significantly associated with TB susceptibility (*p* < 0.05) after adjusting for age, sex and ancestry (**Table 3**), but not after correcting for multiple testing. In this study, we view the methods of correction for multiple testing (including Bonferonni) to be too conservative for this analysis; there is *a priori* evidence that variants in LD with those reported here were associated with TB susceptibility (**Table 1**) [33–35]. For completeness however, nominal *p* values adjusted for multiple testing using the Šidák method are reported in **Table 3**[36].

**Table 2:** Case-control sample characteristics and TB susceptibility modelling results.

	TB Cases (n = 398)	Controls (n = 360)	*p* value*
Age (mean ± SD)	36.55 ± 11.26	30.69 ± 12.80	0.0001
Number of males (proportion)	211 (0.53)	111 (0.28)	< 0.0001
KhoeSan [IQR]	0.30 [0.20–0.39]	0.27 [0.18–0.36]	0.0224
West African [IQR]	0.27 [0.16–0.39]	0.25 [0.15–0.37]	0.3187
European [IQR]	0.18 [0.08–0.28]	0.19 [0.12–0.28]	0.7804
South Asian [IQR]	0.12 [0.03–0.19]	0.14 [0.06–0.22]	0.2767
East Asian [IQR]	0.09 [0.03–0.16]	0.10 [0.05–0.17]	NA^a^

^a^ The East Asian component was not added to the model to avoid linear dependency.

SD standard deviation, IQR, interquartile range

*Statistic is an indication of the significance of the association between each factors with TB after adjusting for the other factors.

**Table 3:** Association results of statistically significant regulatory SNPs.

rsID	Controls		HWE^c^ *p*-val	TB Cases		HWE *p*-val	Association *p*-val Adjusted^d^	Model of penetrance	OR	2.5% CI	97.5% CI	Šidák *p*-val
	Count^a^	Prop^b^		Count	Prop							
**rs2284555 (*IFNGR2*)**												
	352		0.457	390		0.208						
G/G	37	0.11		25	0.06		0.043					0.997
A/G	145	0.41		168	0.43			Genotypic	2.146	1.174	3.988	
A/A	170	0.48		197	0.51			Genotypic	2.012	1.108	3.716	
G	219	0.31		218	0.28							
A	485	0.69		562	0.72		0.179					
**rs829161 (*ACACA*)**												
	348		0.539	387		0.015						
T/T	208	0.6		236	0.61		0.037					0.990
T/C	125	0.36		121	0.31							
C/C	15	0.04		30	0.08			Genotypic	2.274	1.133	4.727	
T	541	0.78		593	0.77		0.292					
C	155	0.22		181	0.23							
**rs7599352 (*MARCO*)**												
	327		0.034	353		0.438						
C/C	183	0.56		213	0.6		0.021					0.941
C/T	113	0.35		126	0.36							
T/T	31	0.09		14	0.04			Genotypic	0.416	0.197	0.842	
C	479	0.73		552	0.78		0.244					
T	175	0.27		154	0.22							
**rs4687614 (*NISCH*)**												
	355		1	391		0.733						
G/G	7	0.02		14	0.04		0.040					
A/G	87	0.25		114	0.29							
A/A	261	0.74		263	0.67							
G	101	0.14		142	0.18							
A	609	0.86		640	0.82		0.012	Additive	1.464	1.089	1.980	0.799
**rs2600665 (*AHSA2*)**												
	350		0.811	383		0.691						
T/T	156	0.45		207	0.54		0.019					
T/G	154	0.44		152	0.4							
G/G	40	0.11		24	0.06							
T	466	0.67		566	0.74		0.006	Additive	0.714	0.559	0.910	0.551
G	234	0.33		200	0.26							
**rs12233670 (*TLR10/1*)**												
	352		0.194	390		0.023						
T/T	105	0.3		136			0.022					
T/C	186	0.53		169				Genotypic	0.699	0.491	0.994	0.948
C/C	61	0.17		85								
T	396	0.56		441			0.815					
C	308	0.44		339								

^a^ Allelic and genotype counts

^b^ Allelic and genotype proportions

^c^ Statistic for the HWE exact test. This was stratified by TB susceptibility status.

^d^ Statistic to indicate the association between genotype and TB susceptibility after adjusting for age, gender and ancestry. The allelic effect was modelled using the additive model.

The SNPs rs7599352, rs2600665, rs829161 and rs12233670 showed statistically significant associations with resistance to TB (nominal *p* = 0.021, 0.006, 0.037 and 0.022, respectively). The minor homozygote (T/T) of rs7599352 was found in fewer cases than controls and the genotype has an odds ratio of 0.416 (95% CI = 0.197–0.842). In contrast, rs2600665 followed an additive model of penetrance and thus for every copy of the G allele, an individual is ~30% less likely to progress to active disease. The ancestral homozygote (C/C) of rs829161 was found in double the amount of cases than controls and was associated with susceptibility to TB (nominal *p* = 0.037). This genotype has an odds ratio of 2.274 (95% CI = 1.13–4.73). The heterozygote genotype of rs12233670 was associated with TB susceptibility and with an odds ratio of 0.699 (95% CI = 0.491–0.994). Two SNPs, namely rs4687614 and rs2284555, were associated with increased susceptibility to TB. For every copy of the minor G allele of rs4687614, there was an increase of ~50% in the likelihood of progressing to active TB (*p* = 0.040, 95% CI = 1.09–1.98). In addition, rs2284555 was associated with TB susceptibility (*p* = 0.043); both the minor homozygote and heterozygote were associated with the phenotype. The minor homozygote has an odds ratio of 2.012 (95% CI = 1.108–3.716) whereas the heterozygote yielded an odds ratio of 2.146 (95% CI = 1.174–3.988).

## Discussion

The genetic factors influencing TB susceptibility have been under investigation for many years, using various study designs, with limited success (S1 Table). This is due to a number of factors, including lack of power, the inability to identify causative variants and the complexity of admixed populations. We conducted a post-GWAS analysis of predicted functional variants and investigated their associations with TB susceptibility in the admixed SAC population. This resulted in the identification of 3 variants associated with an increased risk of progressing to active TB and another 3 variants associated with resistance to active TB.

*MAR*CO (Macrophage Receptor With Collagenous Structure) is a member of the class A scavenger receptor family and has been implicated in innate antimicrobial activity, more specifically TB susceptibility, in the Gambian and Chinese Han populations [38, 39]. Statistically significant variants from Songane et al. (2012) and Bowdish et al. (2013) were used as query SNPs in the study presented here [39, 40]. The rs7599352 variant has not directly been implicated in TB susceptibility previously, but other intronic variants have been shown to have an effect on *MARCO* gene expression [38, 39]. This implies that intronic variants within this gene could have a greater functional effect than exonic variants.

Toll-like receptors (TLRs) are responsible for pathogen recognition and the resulting activation of an innate immune response. TLR genes, including *TLR2*, *TLR4* and *TLR8*, are known to contribute to TB susceptibility [41] and numerous variants within *TLR1* and *TLR10* have also been associated with TB susceptibility in previous studies [42, 43]. We provide evidence for a novel SNP (rs12233670) associated with a decrease in risk of TB susceptibility. This SNP was in LD with statistically significant SNPs reported in Ma et al. (2007) and Uciechowski et al. (2011) [42, 44].

Due to the long-range effects of LD with variants in Dual Specificity Phosphatase 14 (*DUSP14)* and *XPO1* (S1 Table), two novel genes were associated with increased TB susceptibility and increased resistance, namely, Acetyl-CoA Carboxylase Alpha (*ACACA)* and Activator Of Heat Shock 90kDa Protein ATPase Homolog 2 (*ASHA2)* respectively. An intronic variant (rs829161) in the *ACACA* gene was highly associated with the susceptibility phenotype with an odds ratio suggesting more than a 2.2 times increased chance of the disease progressing to an active state than remaining latent. The *ACACA* gene is involved in fatty acid carboxylation and in turn mediates the removal of cholesterol. Cholesterol is used as an energy source for *Mycobacterium tuberculosis (M*.*tb)* therefore any disruption of the level of the fatty acid may influence the dormancy of the bacteria [45]. A possible novel resistance pathway was identified in this study, involving heat shock protein (HSP) activation and resulting ATPase activity by ASHA2. In this case the variant (rs260065) is located in the 5’-untranslated region and we therefore hypothesize that it has a profound regulatory function, potentially affecting gene expression. HSPs play a pivotal role in protein folding, stabilization and degradation, and are targets of chemotherapy in cancer patients as HSP modulates tumour cell apoptosis through protein kinase B, tumour necrosis factor receptor and NF-kB functioning. Due to the interaction with these known immune response genes, we hypothesize that ASHA2 could be involved in TB immune responses.

A variant (rs4687614) in the Nischarin (*NISCH)* gene was found to be associated with an increase in TB susceptibility. *NISCH* encodes the Nischarin protein. Nischarin has recently been identified as a dual effector that interacts with members of the Rac and Rab GTPase families [46]. The regulation of GTPases by Nischarin may regulate the maturation and acidification of vacuoles that are associated with phagocytosis of bacterial pathogens [46]. The odds ratio associated with the variant indicated an increased risk of progressing to active TB. An association was also found for a polymorphism in the Interferon-gamma Receptor 2 (*IFNGR2)* gene. An intronic variant (rs2284555) yielded a high odds ratio of 2.01 for the A/A genotype and 2.15 for the A/G genotype. *IFNGR2* has been previously implicated in various immunodeficiencies [47–50]. This study reiterates the role of IFNGR2 in antibacterial immune response and provides a novel causative SNP for TB.

Only 6 out of a total of 133 SNPs genotyped produced statistically significant associations with TB susceptibility. The predicted regulatory SNPs genotyped in our study were in strong LD with variants previously associated with TB in other populations. A comparison between the *p*-values of the original GWAS SNPs and the six variants identified here, shows that the *p*-values are lower in the original GWAS data. This could be an artefact due to the low sample size in our study, an increase in error variation or the moderate to low effect sizes associated with the six variants [51]. Susceptibility to TB is a complex disease and it is possible that numerous small/moderate effect variants at a frequency less than 0.05 will play a role in this phenotype [52]. It is also possible that some of the variants identified by previous studies are population-specific susceptibility variants and are therefore unlikely to be involved in disease predisposition in the SAC population. One might have expected many more associations due to the strong LD between SNPs genotyped in our study and variants previously identified as being associated with TB. The lack of associations suggest that these SNPs are not the causative SNPs that led to significant results in previous studies and that other SNPs in LD with the marker variants may play a role. Alternatively, the variants have smaller effect sizes than we could detect with our sample size. However, the power to detect a common variant with an odds ratio of 2 was 98%. Validation of these results in other case-control cohorts as well as the inclusion of recent GWAS results [5,12,14] is desirable, but complicated by the lack of available TB case-control cohorts with a similar genetic structure to that of the SAC population. In addition, since there was *a priori* evidence for an association, replication is arguably not necessary as this study attempted to fine-map the potential causal variants in loci identified by previous TB GWAS.

Age, KhoeSan ancestry and sex differed significantly between the TB cases and controls in this study, but were adjusted for in all analyses. We have previously shown that KhoeSan ancestry increases the risk of active TB [32] and old age is also a known TB risk factor [53]. Based on our experience in these communities, healthy males are less likely to attend clinics, while healthy females will accompany sick children and are therefore more likely to participate as controls. However, in most countries the TB notification rate is twice as high in males as in females [54] and evidence suggests that the X chromosome does play a role in TB susceptibility [55]. An investigation of sib-pair families from The Gambia indicated that chromosome Xq might be involved in TB susceptibility [55]. Additionally, sex-specific TB associations for *TLR8*, an X-linked gene, has been identified in several populations [56–58], including the SAC population [59]. These loci could contribute to the observed male sex-bias in this population, but will require further investigation.

Subsequent to the analysis presented here, Chimusa et al. (2016) published a post-GWAS methodology utilizing LD information and the human protein–protein interaction network, which identified novel pathways associated with breast cancer [52]. The study by Chimusa et al. (2016) reiterates the need for alternative methodologies in the identification of regulatory variants associated with a phenotype.

In summary, the six predicted functional variants associated with TB susceptibility in the SAC population show that fine mapping of GWAS results can reveal candidate causal variants. Functional analyses are now required to elucidate the molecular mechanisms by which these polymorphisms may act, while well-powered TB GWAS and meta-analyses may continue to identify additional causal variants for TB susceptibility.

## Supporting information

S1 Table Variants associated with TB susceptibility. This table represents the variants that have been implicated in TB susceptibility by association studies. The closest gene to the variant is reported.
(ZIP)

S2 Table The functional impact of proxy SNPs as determined by RegulomeDB. A score of 1 is the lowest score possible as determined by RegulomeDB. This indicates strong evidence of a functional impact. This has been determined by a variety of comprehensive datatypes.
(ZIP)
